# The association between hemoglobin A1c and all-cause mortality in the ICU: A cross-section study based on MIMIC-IV 2.0

**DOI:** 10.3389/fendo.2023.1124342

**Published:** 2023-02-15

**Authors:** Chunxia Liu, Ke Pang, Jianbin Tong, Wen Ouyang, Liang Li, Yongzhong Tang

**Affiliations:** ^1^ Department of Anesthesiology, Third Xiangya Hospital, Central South University, Changsha, China; ^2^ Department of Gastrointestinal Surgery, Third Xiangya Hospital, Central South University, Changsha, China

**Keywords:** hemoglobin A1c, mortality, the Medical Information Mart for Intensive Care, diabetes, cross-sectional study

## Abstract

**Background:**

Hyperglycemia has been reported to be associated with the outcomes of patients in the intensive care unit (ICU). However, the relationship between hemoglobin A1c (HbA1c) and long-term or short-term mortality in the ICU is still unknown. This study used the Medical Information Mart for Intensive Care (MIMIC)-IV database to investigate the relationship between HbA1c and long-term or short-term mortality among ICU patients without a diabetes diagnosis.

**Methods:**

A total of 3,154 critically ill patients without a diabetes diagnosis who had HbA1c measurements were extracted and analyzed from the MIMIC-IV. The primary outcome was 1-year mortality, while the secondary outcomes were 30-day mortality and 90-day mortality after ICU discharge. HbA1c levels were classified into four levels according to three HbA1c values (5.0%, 5.7%, and 6.5%). The Cox regression model was used to investigate the relationship between the highest HbA1c measurement and mortality. Finally, this correlation was validated using the XGBoost machine learning model and Cox regression after propensity score matching (PSM).

**Results:**

The study eventually included 3,154 critically ill patients without diabetes who had HbA1c measurements in the database. HbA1c levels of below 5.0% or above 6.5% were significantly associated with 1-year mortality after adjusting for covariates in Cox regression (HR: 1.37; 95% CI: 1.02–1.84 or HR: 1.62; 95% CI: 1.20–2.18). In addition, HbA1c 6.5% was linked to 30-day mortality (HR: 1.81; 95% CI: 1.21–2.71) and 90-day mortality (HR: 1.62; 95% CI: 1.14–2.29). The restricted cubic spline demonstrated a U-shaped relationship between HbA1c levels and 1-year mortality. The AUCs of the training and testing datasets in the XGBoost model were 0.928 and 0.826, respectively, while the SHAP plot revealed that HbA1c was somewhat important for the 1-year mortality. Higher HbA1c levels in Cox regression were still significantly associated with 1-year mortality after PSM for other factors,

**Conclusions:**

The 1-year mortality, 30-day mortality, and 90-day mortality rates for critically ill patients after discharge from ICU are significantly associated with HbA1c. HbA1c < 5.0% and ≥6.5% would increase 30-day, 90-day, and 1-year mortality, while levels between 5.0% and 6.5% of HbA1c did not significantly affect these outcomes.

## Background

Currently, the mortality rate of critically ill patients in intensive care units (ICUs) remains high. According to previous studies, the global ICU mortality rate is approximately 15.5%–16.9% (1), and the ICU mortality rate in France is approximately 15% (2), while the ICU mortality rate in Norway is approximately 12.7% and 30-day mortality is approximately 21.2% (3). ICU or in-hospital mortality is significant; moreover, mortality following ICU discharge also has a significant impact on critically ill patients’ quality of life (4, 5). To treat the patients’ varied complications, several actions will be taken.

Even if these patients were previously normoglycemic, stress-induced hyperglycemia in an ICU is a typical consequence (6). In ICUs, stress and hyperglycemia are related. High quantities of counter-regulatory hormones such as glucagon, adrenaline, cortisol, and growth hormone are released in response to stress in the ICU, promoting hepatic gluconeogenesis and insulin resistance, which result in hyperglycemia (7, 8). Insulin resistance inhibits insulin-mediated glucose transport. Thus, in the internal environment, there is less glucose consumption and more gluconeogenesis, resulting in hyperglycemia (9). Hyperglycemia occurs more frequently in critical settings (10). According to a study, hyperglycemia is a sign of serious illness or trauma and a stand-alone predictor of poor outcomes in critically ill patients in the ICU (11, 12). Elevated blood sugar levels led to prolonged ICU and in-hospital stay and a higher incidence of infection or death (13, 14).

Previous studies suggested that hyperglycemia or hemoglobin A1c (HbA1c) might be associated with a poor prognosis in critically ill patients without diabetes (15). Several studies have found that HbA1c predicts ICU mortality in COVID-19 severe pneumonia, with patients with severe COVID-19 pneumonia having a higher 28-day mortality (16). Furthermore, compared to COVID-19 patients with a diabetes diagnosis, critically ill and COVID-19 patients with HbA1c levels above 6.5% and no prior diagnosis of diabetes had the highest risk of all-cause mortality (17). Furthermore, there was a study reporting that critically ill patients with unknown diabetes had higher mortality and higher glycemic variability (18), which were reported to be independently associated with in-hospital mortality and 30-day mortality in the ICU (19, 20). The relationship between HbA1c and long- or short-term outcomes following ICU discharge, on the other hand, has not been studied. One study showed that in-hospital control of glucose parameters reduced the length of stay and decreased 30-day and 1-year mortality in critically ill patients in cardiac ICU (21).

Based on the MIMIC-IV database and 3,154 patients without a diabetes diagnosis, this study assessed the relationship between the highest hypoglycemia A1c during ICU stay and long- and short-term outcomes after discharge.

## Methods

### Data source

This research was performed on a large, free, and public database, namely, Medical Information Mart for Intensive Care (MIMIC)-IV (22, 23), which comprised comprehensive clinical information on hospital stays for patients admitted to a tertiary academic medical center in Boston. The latest version, MIMIC-IV 2.0, was updated in June 2022 and contains comprehensive information about patients. The structured query language (SQL) was used for data extraction from the MIMIC-IV database.

### Population selection criteria

**Figure 1:**
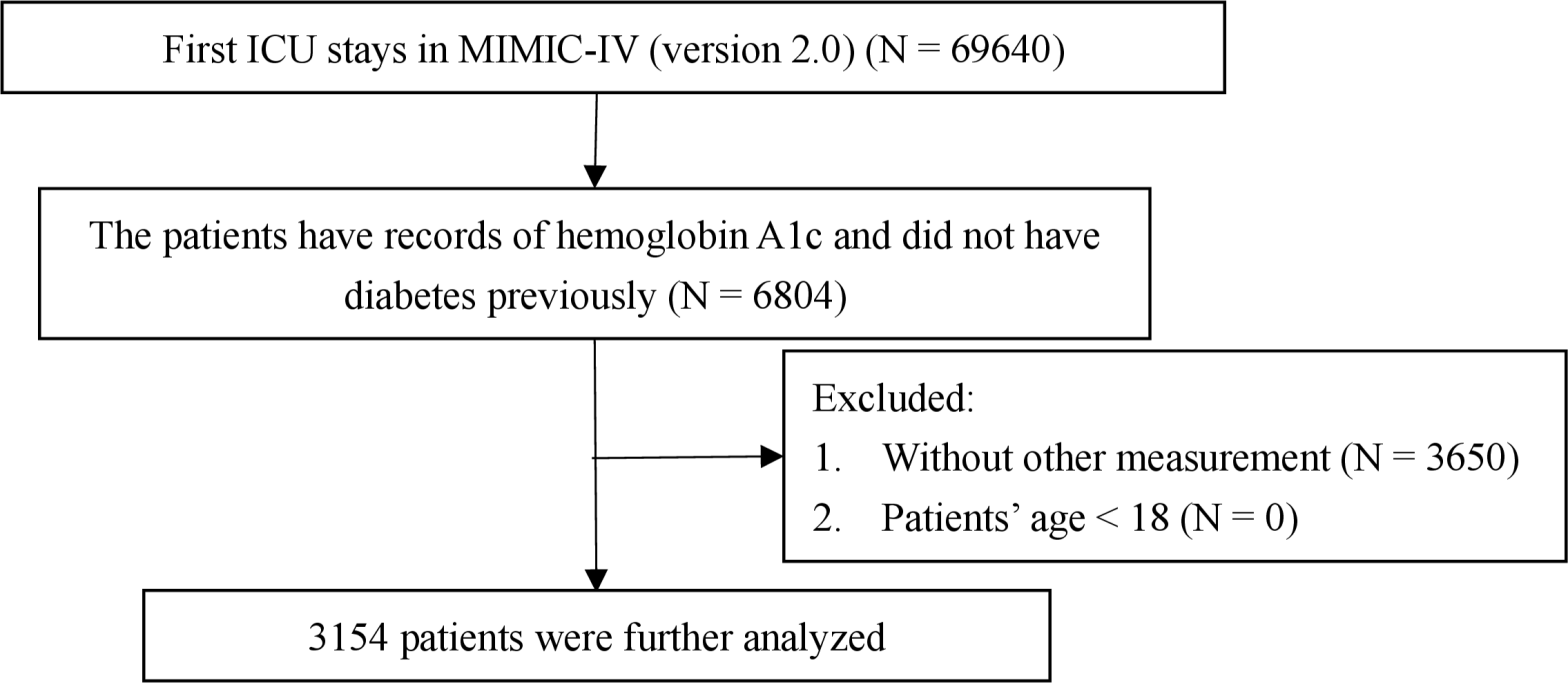
Flowchart of study population selection.

The inclusion criteria included the following: patients admitted into the ICU for the first time, HbA1c was measured at least once, and there had been no previous diabetes diagnosis. The exclusion criteria were as follows: no height or other measurements and patients under the age of 18 (**Figure 1**).

### Outcomes and covariates

The primary outcome was long-term (1 year) mortality. The secondary outcomes included short-term (30- and 90-day) mortality. The extraction variables included age, gender, body mass index (BMI), admission type, congestive heart failure, renal disease, hypertension, acute myocardial infarction (AMI), creatinine, blood urea nitrogen (BUN), hemoglobin, the Sequential Organ Failure Assessment (SOFA) score, the Simplified Acute Physiology Score (SAPS II), HbA1c, reintubation event, accumulated ventilation time, renal replacement therapy, the heart surgery, the use of some drugs [including aspirin, beta blockers, angiotensin-converting enzyme inhibitors (ACEIs), angiotensin II receptor blockers (ARBs), dopamine, dobutamine, norepinephrine, and vasopressin]. In addition, HbA1c measurements were divided into four groups, namely, <5.0 mmol/L, 5.0–5.7 mmol/L, 5.7–6.5 mmol/L, and ≥ 6.5 mmol/L.

### Statistical analyses

Categorical data were expressed as percentages and frequencies, while continuous data were expressed as mean SD or median (range) values. Cox proportional hazards regression models were used to calculate the hazard ratios (HRs) and their corresponding 95% confidence intervals (CIs) for 30-day, 90-day, and 1-year mortality. Model I was not adjusted, whereas Model II was adjusted for age, BMI, gender, admission type, congestive heart failure, renal disease, hypertension, acute myocardial infarction, creatinine, BUN, hemoglobin, and the SOFA score. After adjusting with Cox analysis regression using other covariates, restricted cubic splines were used to confirm the linearity between HbA1c and outcome. Using propensity score matching (PSM), the surviving patients were matched with patients of the same size from the deceased. A univariate Cox regression analysis was used to analyze the correlation between HbA1c and outcome. In addition, the XGBoost (24) machine learning algorithm was used to validate the significance of HbA1c on 1-year mortality outcomes. ROC curves were also plotted to confirm the prediction ability of the model, and SHAP figures were plotted to validate the importance of HbA1c on outcomes across all these variables.

R (version 4.1.0) software was used for the statistical analysis. *p* < 0.05 was considered statistically significant.

## Results

The study introduced a total of 3,154 patients without diabetes who had HbA1c measurements from the MIMIC-IV database; 581 of those patients were deceased in 365 days, while 2,573 of them survived (**Table 1**). Patients in the death group were older than those in the survival group, and they also had higher BUN and creatinine measurements, and lower hemoglobin measurements. Moreover, the death group had higher SOFA scores, higher SPASII scores, longer ventilation times, and higher proportion of congestive heart failure, hypertension, renal disease, renal replacement therapy, and reintubation events.

**Table 1:** Characteristics of the study population (*N* = 3,154).

	Survival	Dead	*p*
N	2573	581	
Age ^1^ (mean (SD))	65.70 (14.81)	73.80 (15.19)	<0.001
Blood urea nitrogen ^1^ (mean (SD))	28.20 (19.51)	44.89 (32.46)	<0.001
Creatinine ^1^ (mean (SD))	1.39 (1.36)	1.93 (1.60)	<0.001
Hemoglobin ^1^ (mean (SD))	9.54 (2.45)	8.92 (2.45)	<0.001
SOFA score ^1^ (mean (SD))	4.39 (3.12)	6.16 (3.76)	<0.001
SAPSII score ^1^ (mean (SD))	32.22 (11.59)	41.82 (12.52)	<0.001
Ventilation time ^1^ (mean (SD))	875.93 (2,701.94)	2,594.65 (5,554.92)	<0.001
Gender = Male ^2^ (%)	1,666 (64.7)	293 (50.4)	<0.001
Congestive heart failure^2^ (%)	677 (26.3)	203 (34.9)	<0.001
Renal disease^2^ (%)	311 (12.1)	146 (25.1)	<0.001
Hemoglobin A1c ranges^2^ (%)			<0.001
<5.0%	176 (6.8)	60 (10.3)	
5.0% ≤ HbA1c < 5.7%	1,228 (47.7)	236 (40.6)	
5.7% ≤ HbA1c < 6.5%	1,022 (39.7)	227 (39.1)	
6.5% ≤ HbA1c	147 (5.7)	58 (10.0)	
Hypertension^2^ (%)	1,732 (67.3)	429 (73.8)	0.003
Acute myocardial infarction^2^ (%)	536 (20.8)	84 (14.5)	0.001
Use of aspirin^2^ (%)	2,003 (77.8)	335 (57.7)	<0.001
Use of beta blocker^2^ (%)	1,891 (73.5)	369 (63.5)	<0.001
Use of ACEI^2^ (%)	881 (34.2)	127 (21.9)	<0.001
Use of ARB^2^ (%)	231 (9.0)	34 (5.9)	0.018
Use of dopamine^2^ (%)	46 (1.8)	25 (4.3)	<0.001
Use of dobutamine^2^ (%)	40 (1.6)	25 (4.3)	<0.001
Use of norepinephrine^2^ (%)	356 (13.8)	152 (26.2)	<0.001
Use of vasopressin^2^ (%)	122 (4.7)	82 (14.1)	<0.001
Admission type^2^ (%)			<0.001
Scheduled surgery	228 (8.9)	14 (2.4)	
Medical admission	1,738 (67.5)	462 (79.5)	
Unscheduled surgery	607 (23.6)	105 (18.1)	
Reintubation event^2^ (%)	325 (12.6)	108 (18.6)	<0.001
Renal replacement therapy^2^ (%)	63 (2.4)	41 (7.1)	<0.001

Data are number of subjects (percentage) or mean (standard derivatives); ^1^ One-way ANOVA test was used to compare the mean ± standard deviance values between deceased and survived participants; ^2^ Chi-square test was used to compare the percentage between deceased and survived participants.

### Restricted cubic splines

**Figure 2:**
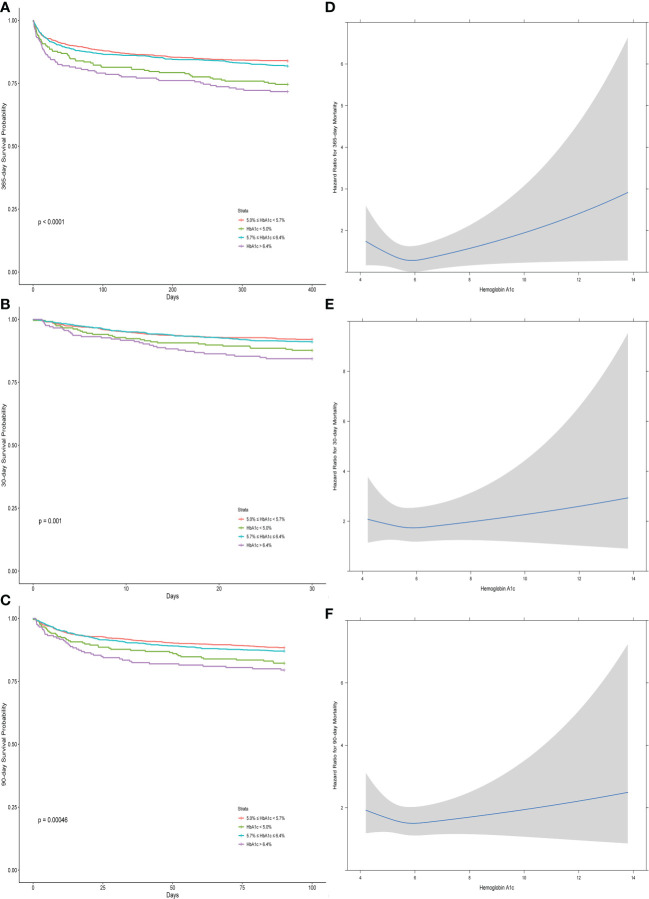
The Kaplan–Meier curves **(A–C)** and the restricted cubic splines **(D–F)** between the hemoglobin A1c and different outcomes (30-day, 90-day, and 1-year mortality).

**Figures 2A–C** depict the restricted cubic spline (after adjusting with Cox analysis regression), with **Figure 2A** revealing the U-shaped association between HbA1c and 1-year mortality, while **Figures 2B**, **C** did not reveal the U-shaped association between HbA1c and 30-day and 90-day mortality and only revealed a negative association between mortality and HbA1c in the range of 5% and 6%.

**Figures 2D–F** show the differences between groups with different ranges of HbA1c for different outcomes, which revealed that the medium ranges of HbA1c (5.0%–5.7% and 5.7%–6.4%) had relatively higher 1-year survival rate than the remaining ranges of HbA1c (less than 5.0% and more than 6.4%). Only the groups with the highest HbA1c (more than 6.4%) had a significantly higher deceased rate for the 30-day and 90-day mortality.

### Univariate and multivariate analyses

The Cox model showed that different levels of HbA1c were associated with the occurrence of long-term (1-year) mortality, as well as short-term (30-day and 90-day) mortality when unadjusted and adjusted in **Table 2**. In the univariate Cox model, those who reported an HbA1c level of less than 5 mmol/L had an HR of 1.65 (95% CI, 1.24–2.19), and those with more than 6.4 mmol/L had an HR of 1.89 (95% CI, 1.42–2.53), compared with the normal range (5.0–5.7 mmol/L). After adjusting for gender, age, admission type, congestive heart failure, renal disease, acute myocardial infarction, creatinine, BUN, hemoglobin, SOFA scores, SAPSII scores, ventilation time, the use of drugs including aspirin, beta blockers, ACEI, ARB, dopamine, dobutamine, norepinephrine, and vasopressin, renal replacement therapy, and heart surgery, compared with the normal range of HbA1c (5.0–5.7 mmol/L), the lower range (less than 5.0 mmol/L) and the higher range (more than 6.4 mmol/L) had HRs of 1.37 (95% CI, 1.02–1.84) and 1.62 (95% CI, 1.20–2.18), respectively. For the 30-day mortality outcome, in the univariate Cox model, those who reported an HbA1c level of less than 5 mmol/L had an HR of 1.58 (95% CI, 1.05–2.38), and those with more than 6.4 mmol/L had an HR of 2.05 (95% CI, 1.39–3.03), while in the adjusted model, the higher level of HbA1c (more than 6.5%) had an HR of 1.81 (95% CI, 1.21–2.71). For the 90-day mortality outcome, in the univariate Cox model, those who reported an HbA1c level of less than 5 mmol/L had an HR of 1.58 (95% CI, 1.13–2.22), and those with more than 6.4 mmol/L had an HR of 1.87 (95% CI, 1.33–2.62), and in the adjusted model, the higher range of HbA1c (more than 6.5%) had an HR of 1.62 (95% CI, 1.14–2.29).

**Table 2:** Univariate and multivariate analysis of the associations between outcomes and different ranges of hemoglobin A1c.

	Group 1 (5.0% ≤ HbA1c < 5.7%)	Group 2 (HbA1c < 5.0%)		Group 3 (5.7% ≤ HbA1c < 6.5%)		Group 4 HbA1c≥6.5%)	
	HR (95% CI)	HR (95% CI)	*p*-value	HR (95% CI)	*p*-value	HR (95% CI)	*p*-value
Primary outcome							
1-year mortality							
Unadjusted	Reference	1.65 (1.24–2.19)	<0.001	1.13 (0.95–1.36)	0.176	1.89 (1.42–2.53)	<0.001
Adjusted	Reference	1.37 (1.02–1.84)	0.038	1.08 (0.90–1.30)	0.412	1.62 (1.20–2.18)	0.001
Second outcome							
30-day mortality							
Unadjusted	Reference	1.58 (1.05–2.38)	0.027	1.12 (0.86–1.45)	0.392	2.05 (1.39–3.03)	<0.001
Adjusted	Reference	1.37 (0.89–2.12)	0.150	1.13 (0.87–1.48)	0.364	1.81 (1.21–2.71)	0.004
90-day mortality							
Unadjusted	Reference	1.58 (1.13–2.22)	0.008	1.12 (0.90–1.39)	0.297	1.87 (1.33–2.62)	<0.001
Adjusted	Reference	1.33 (0.93–1.91)	0.113	1.10 (0.88–1.37)	0.416	1.62 (1.14–2.29)	0.007

### Propensity score matching

**Figure 3:**
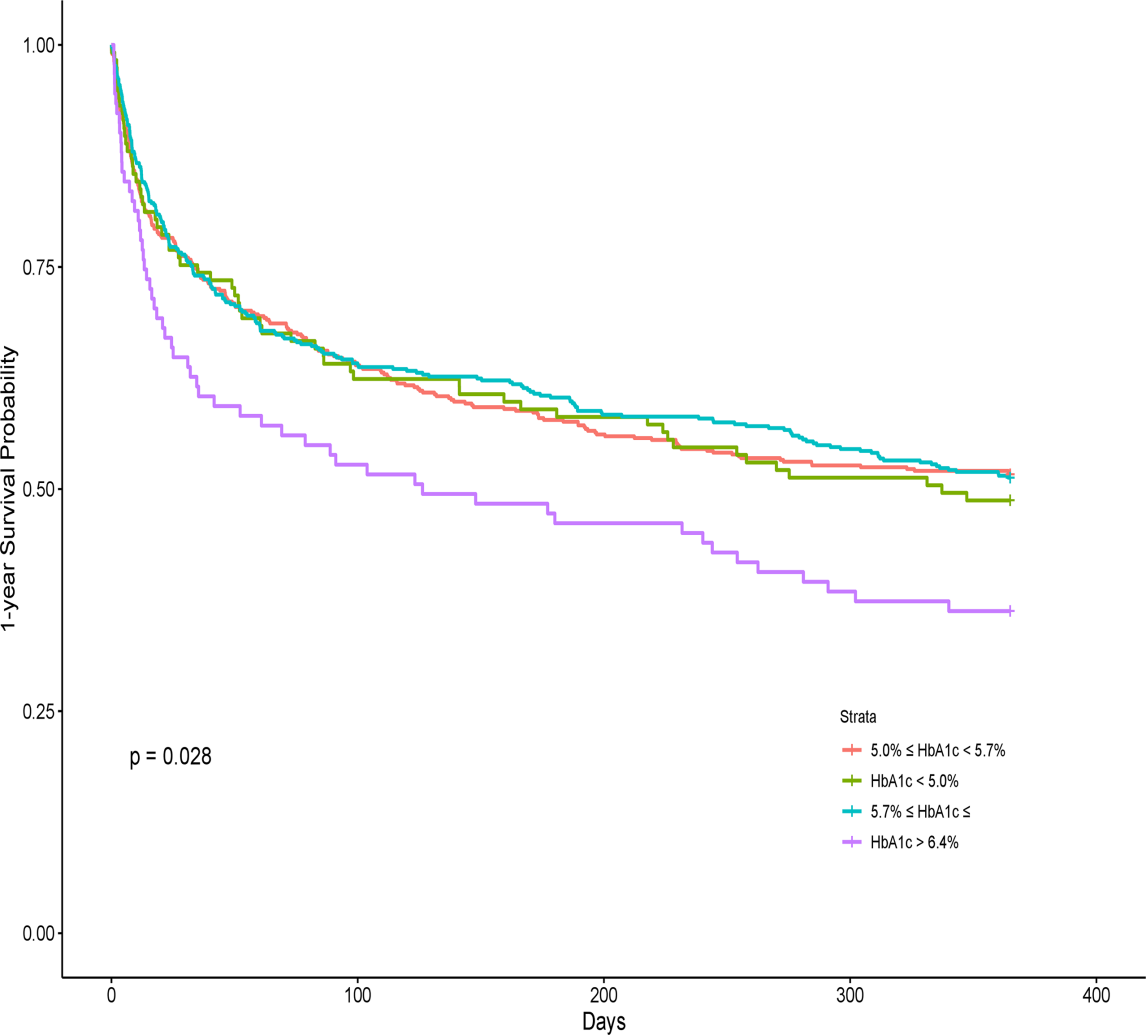
The Kaplan–Meier curve between the hemoglobin A1c and 1-year mortality after PSM.

After PSM, the whole sample size included 581 deceased patients and 581 survived patients (**Supplementary Table 1**). The mirror histogram in **Supplementary Figure 1** described the propensity score distributions in the survived and deceased groups before and after PSM, respectively. In the univariate Cox regression analysis (**Supplementary Table 2**), only the higher level (more than 6.4%) of HbA1c was significantly associated with 1-year mortality, with an HR of 1.51 (95% CI, 1.13–2.01), which is shown in **Figure 3**.

### XGBoost machine learning algorithm

**Figure 4:**
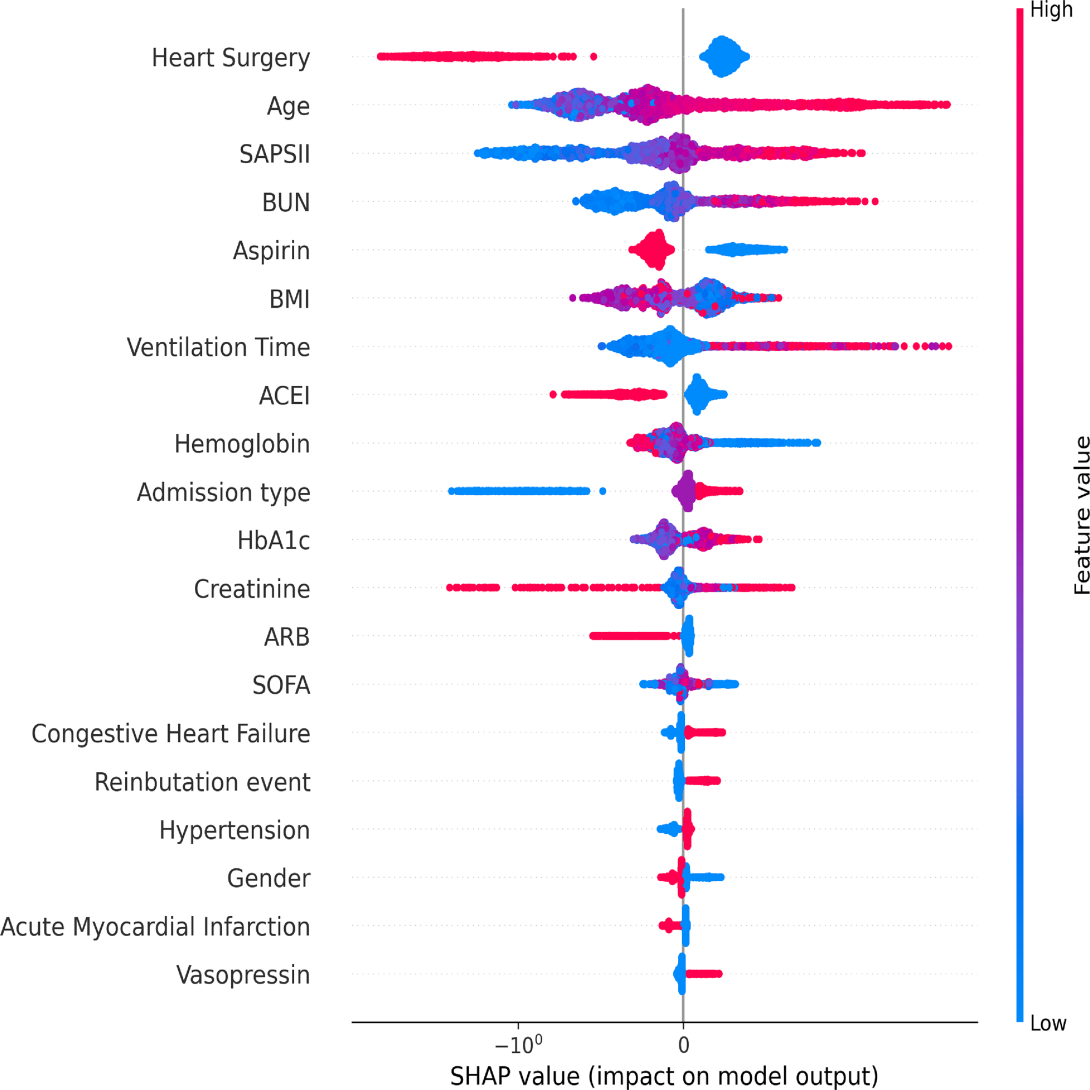
The SHAP values of variables in the XGBoost machine learning model.

**Figure 5:**
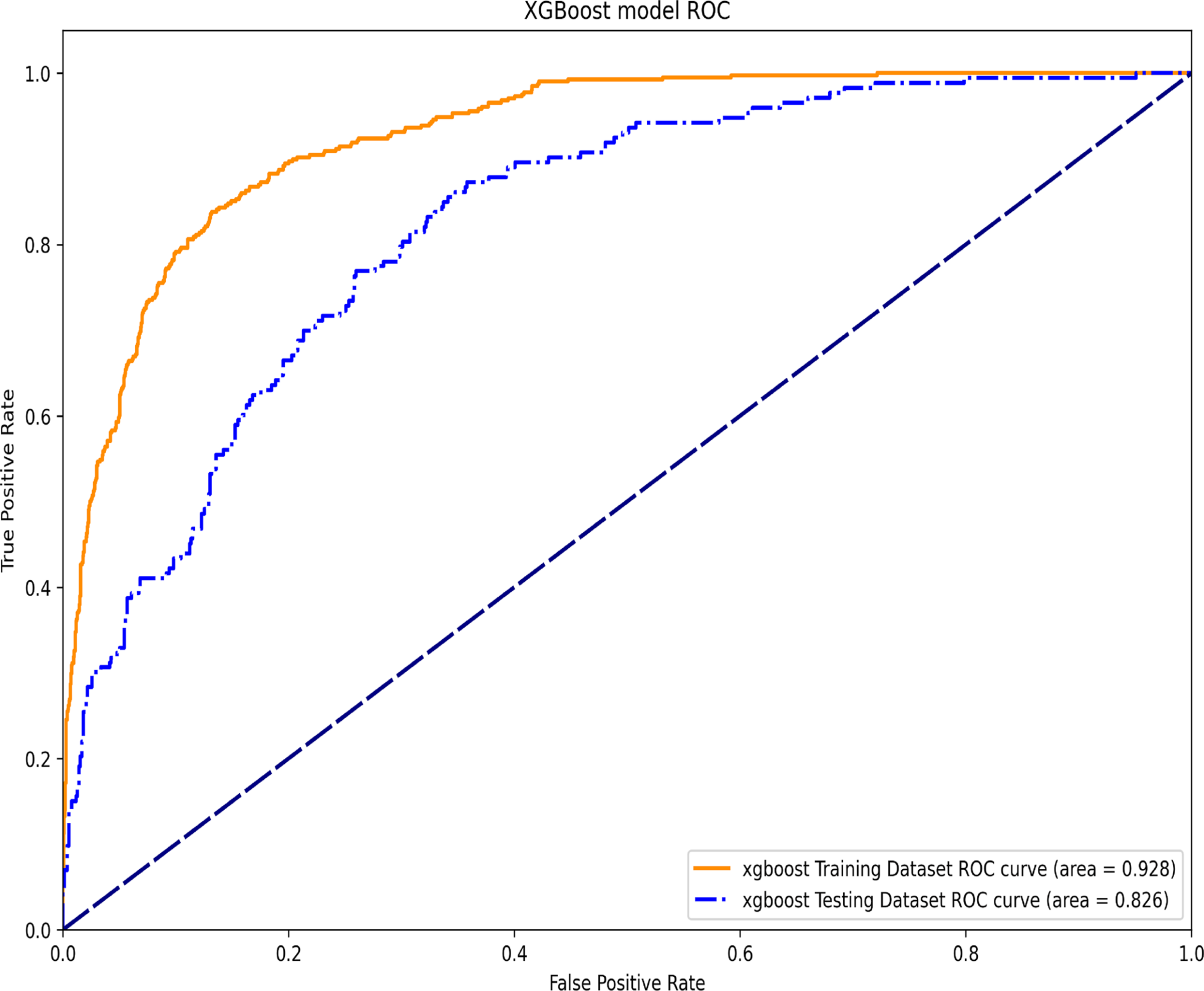
The ROC of the training dataset and the testing dataset in the XGBoost machine learning model.

In this machine learning model, the SHAP figure is shown in **Figure 4**, which showed that, to some extent, HbA1c was important for the 1-year mortality. The ROCs of the training dataset and testing dataset are plotted in **Figure 5**, which showed great prediction ability for the 1-year mortality.

## Discussion

It has been reported that about one in five ICU survivors would pass away within a year (25) and another study found that the 1-year mortality rate among ICU survivors was approximately 20% (26), which was close to the result in our study. Furthermore, despite the absence of a prior diabetes diagnosis, 46.1% of critically ill patients developed hyperglycemia, with a maximum HbA1c greater than 5.7 mmol/L during their hospitalization. The patients with HbA1c above 6.4% and without diabetes were previously considered to have unknown diabetes, which was associated with various adverse outcomes (18, 27).

Our investigation suggested that HbA1c was associated with 1-year, 30-day, and 90-day mortality among patients without diabetes. The SHAP figure can explain the importance of each variable in this model (28). Through SHAP, we could conclude that HbA1c, to some extent, could predict ICU mortality. According to Lee et al., glycated hemoglobin could predict organ dysfunction in critically ill patients with sepsis and might be a predictor for ICU outcomes (29). Another study found that the risk of death doubled with each increase in HbA1c level, and mortality in the critically ill patients was also affected by the chronic hyperglycemia (30). Furthermore, one study showed that, in critically ill patients without diabetes previously, HbA1c at admission was significantly associated with mortality in ICU (31).

In addition to the ICU research mentioned above, some studies found that among 1,474 non-diabetic patients undergoing cardiac surgery, patients with HbA1c above 6% had a significantly higher 30-day mortality outcome than patients with HbA1c below 6% (32). Furthermore, Halkos et al. discovered a significant reduction in long-term survival per unit increase in HbA1c of 7.0% (33). After multivariable adjustment, a different prospective study discovered that patients with HbA1c above 6.0% had significantly higher 3-year mortality rates than those with HbA1c levels < 6%.

Additionally, previous studies revealed that HbA1c values have a U-shaped relationship with all-cause mortality, showing that both high and low HbA1c values are associated with higher mortality rates in elderly patients with acute VTE (34). Moreover, a U-shaped relationship between HbA1c levels and mortality among diabetics receiving hemodialysis was also discovered in another study (35). In one meta-analysis, participants with diabetes showed a U-shaped association between HbA1c and all-cause mortality while patients without diabetes showed a reversed J-shaped association (36). However, according to the Atherosclerosis Risk in Communities study, glycated hemoglobin was related to all-cause mortality in a J-shaped pattern (37). Higher postprandial glucose levels associated with IGT directly elevate HbA1c, which is directly correlated with glycemia (38). Furthermore, the amyotrophic lateral sclerosis mortality risk was twice as high for those with HbA1c levels higher than 6.5% at baseline, compared to those with good HbA1c levels (5.7%) (39). Furthermore, other studies only found a positive association between HbA1c levels and mortality in different population cohorts (40–42), which was somewhat similar to our research result.

Moreover, higher HbA1c levels indicated higher glucose variability (18), which was associated with in-hospital or in-ICU mortality, even in patients with tight glycemic control but high glycemic variability (43). Furthermore, according to Eirini., HbA1c was an independent risk factor for nosocomial infections in critically ill patients (44), a major cause of morbidity and mortality in critically ill patients (45). In addition, there was a review analysis with the conclusion that regardless of the previous diabetic status, HbA1c is a strong predictor of mortality and morbidity (46).

However, there was also a research (47) that revealed that among diabetes patients undergoing cardiac surgery, there was no significant difference between the high-level HbA1c group and the control group, but because this research introduced only 101 patients, this conclusion could not be strongly supported.

The strengths of this study rest on several aspects. Firstly, we chose patients without diabetes from the most recent, complex, and comprehensive MIMIC-IV database (version 2.0). Secondly, we selected a sufficient number of participants for this research, which may have made drawing a conclusion safer. Thirdly, we studied the association between 1-year mortality and HbA1c, which was rarely studied.

However, there were also some limitations in this research. First of all, as a single-center retrospective study with a limited sample size, selection bias exists. Secondly, the sample sizes in various groups with different ranges of HbA1c were not balanced, which contributed to the wide range of the CI of the restricted cubic spline’s second half. Thirdly, the research lacked prospective studies and mechanism studies, which need to be studied further.

## Conclusion

HbA1c (≥6.5%) was associated with harmful effects on the 30-day, 90-day, and 1-year mortality among critically ill patients with undiagnosed diabetes after discharge from the ICU. Measurements on lowering glucose variability should be taken in clinical settings to control hemoglobin A1c.

## Data availability statement

The data that support the findings of this study are available from https://physionet.org/content/mimiciv/2.0/ but restrictions apply to the availability of these data, which were used under license for the current study, and so are not publicly available. Data are however available from the authors upon reasonable request and with permission of PhysioNet.

## Ethics statement

Patient consent was waived because the study was an analysis of a third-party anonymized publicly available database.

## Author contributions

WO and YT designed the study. CL and KP organized data. CL and YT analyzed data and wrote the first draft of the manuscript. JT, KP, and LL revised the manuscript. All authors contributed to the article and approved the submitted version.

## Supplementary material

Supplementary Table 1 Characteristics of the study population after PSM (N = 1162).

Supplementary Table 2 Univariate Analysis of the associations between outcomes and different ranges of hemoglobin A1c after PSM.
