# Supplementary figures and images for: The association between hemoglobin A1c and all-cause mortality in the ICU: A cross-section study based on MIMIC-IV 2.0
Source: Front Endocrinol (Lausanne). 2023 Feb 15;14:1124342. doi: 10.3389/fendo.2023.1124342 (PMC9975393; doi:10.3389/fendo.2023.1124342)

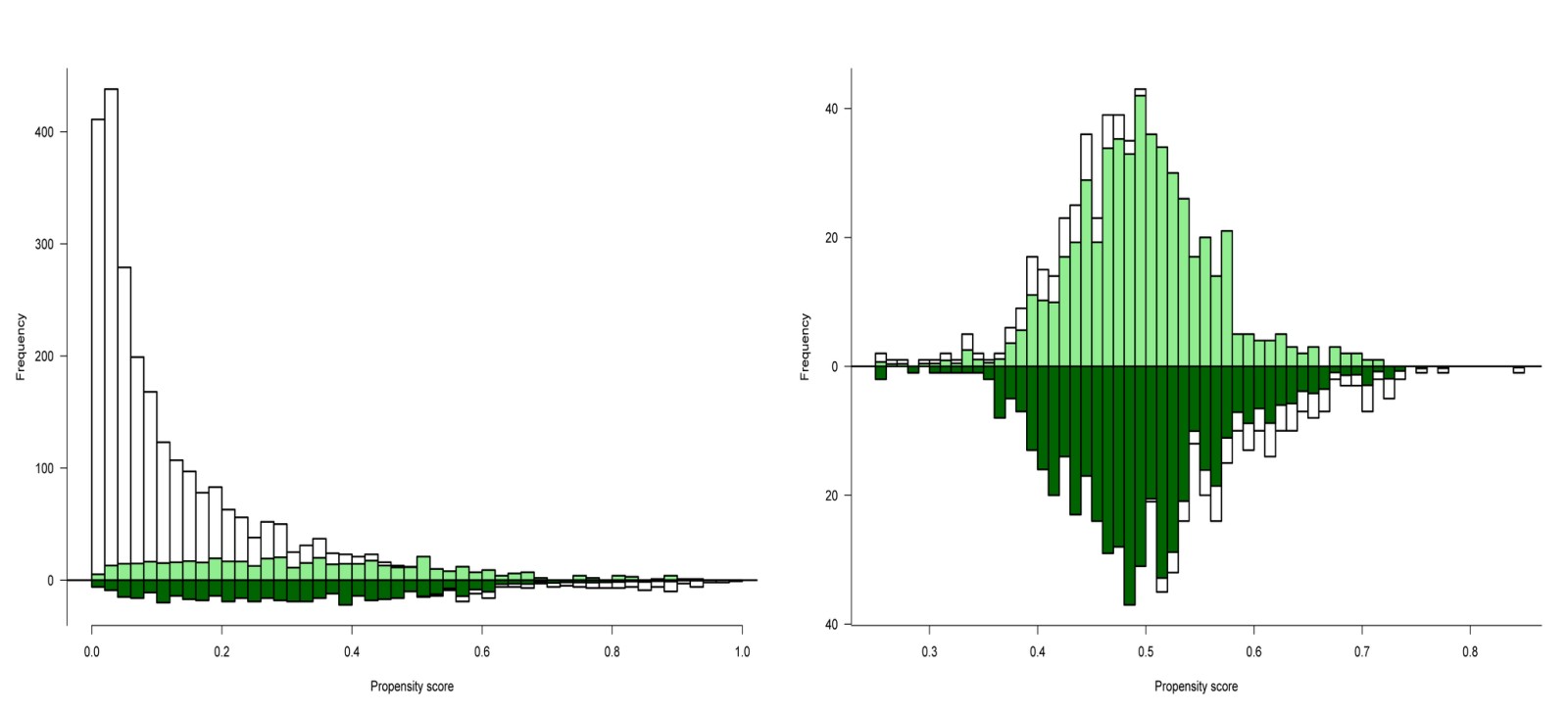

Supplement: Supplementary file 1 [file Image_1.jpg]
