# Supplementary material for: The association between hemoglobin A1c and all-cause mortality in the ICU: A cross-section study based on MIMIC-IV 2.0
Source: Front Endocrinol (Lausanne). 2023 Feb 15;14:1124342. doi: 10.3389/fendo.2023.1124342 (PMC9975393; doi:10.3389/fendo.2023.1124342)
Supplement: Supplementary Table 1 — Characteristics of the study population after PSM (N = 1162). [file Table_1.docx]

**Supplement Table 1** Characteristics of the study population after PSM (N = 1162)

|  | **Survival** | **Dead** | ***p*** |
| --- | --- | --- | --- |
| **N** | 581 | 581 |  |
| **Age (mean (SD))** | 73.44 (12.65) | 73.80 (15.19) | 0.664 |
| **Blood Urea Nitrogen (mean (SD))** | 39.42 (29.04) | 44.89 (32.46) | 0.002 |
| **Creatinine (mean (SD))** | 1.77 (1.83) | 1.93 (1.60) | 0.131 |
| **Hemoglobin (mean (SD))** | 9.06 (2.31) | 8.92 (2.45) | 0.319 |
| **SOFA score (mean (SD))** | 5.61 (3.76) | 6.16 (3.76) | 0.012 |
| **SAPSII score (mean (SD))** | 39.32 (11.87) | 41.82 (12.52) | <0.001 |
| **Ventilation Time (mean (SD))** | 2017.53 (4695.06) | 2594.65 (5554.92) | 0.056 |
| **Gender = Male (%)** | 300 (51.6) | 293 (50.4) | 0.725 |
| **Congestive Heart Failure (%)** | 201 (34.6) | 203 (34.9) | 0.951 |
| **Renal Disease (%)** | 135 (23.2) | 146 (25.1) | 0.493 |
| **Hemoglobin A1c ranges (%)** |  |  | 0.051 |
| < 5.0 % | 57 (9.8) | 60 (10.3) |  |
| 5.0 % ≤ HbA1c < 5.7 % | 252 (43.4) | 236 (40.6) |  |
| 5.7 % ≤ HbA1c < 6.5 % | 239 (41.1) | 227 (39.1) |  |
| 6.5 % ≤ HbA1c | 33 (5.7) | 58 (10.0) |  |
| **Hypertension (%)** | 432 (74.4) | 429 (73.8) | 0.893 |
| **Acute myocardial infarction (%)** | 68 (11.7) | 84 (14.5) | 0.192 |
| **Use of Aspirin (%)** | 351 (60.4) | 335 (57.7) | 0.371 |
| **Use of Beta blocker (%)** | 376 (64.7) | 369 (63.5) | 0.714 |
| **Use of ACEI (%)** | 151 (26.0) | 127 (21.9) | 0.114 |
| **Use of ARB (%)** | 35 (6.0) | 34 (5.9) | 1 |
| **Use of Dopamine (%)** | 17 (2.9) | 25 (4.3) | 0.271 |
| **Use of Dobutamine (%)** | 17 (2.9) | 25 (4.3) | 0.271 |
| **Use of Norepinephrine (%)** | 133 (22.9) | 152 (26.2) | 0.22 |
| **Use of Vasopressin (%)** | 73 (12.6) | 82 (14.1) | 0.49 |
| **Admission Type (%)** |  |  | 0.274 |
| Scheduled Surgery | 23 (4.0) | 14 (2.4) |  |
| Medical Admission | 462 (79.5) | 462 (79.5) |  |
| Unscheduled Surgery | 96 (16.5) | 105 (18.1) |  |
| **Reintubation Event (%)** | 92 (15.8) | 108 (18.6) | 0.244 |
| **Renal Replacement Therapy (%)** | 33 (5.7) | 41 (7.1) | 0.4 |
