# Supplementary material for: The association between hemoglobin A1c and all-cause mortality in the ICU: A cross-section study based on MIMIC-IV 2.0
Source: Front Endocrinol (Lausanne). 2023 Feb 15;14:1124342. doi: 10.3389/fendo.2023.1124342 (PMC9975393; doi:10.3389/fendo.2023.1124342)
Supplement: Supplementary Table 2 — Univariate Analysis of the associations between outcomes and different ranges of hemoglobin A1c after PSM. [file Table_2.docx]

**Supplement Table 2** Univariate Analysis of the associations between outcomes and different ranges of hemoglobin A1c after PSM

.

|  | **Group 1 (Normal group)**  **(5.0 % ≤ HbA1c < 5.7%)** | **Group 2**  **(HbA1c < 5.0%)** | | **Group 3**  **(5.7 % ≤ HbA1c < 6.5 %)** | | **Group 4**  **6.5 % ≤ HbA1c)** | |
| --- | --- | --- | --- | --- | --- | --- | --- |
|  | **HR (95% CI)** | **HR (95% CI)** | ***p-value*** | **HR (95% CI)** | ***p-value*** | **HR (95% CI)** | ***p-value*** |
| **Primary outcome** |  |  |  |  |  |  |  |
| **1-year mortality** |  |  |  |  |  |  |  |
| Unadjusted | Reference | 1.06 (0.80-1.40) | 0.68961 | 0.99 (0.82-1.19) | 0.91110 | 1.51 (1.13-2.01) | 0.00491 |
